# After the Fair

**Published:** 1907-03-23

**Authors:** 


### AFTER THE FAIR.

### A CENTRAL HOSPITAL BOARD ADVOCATED.

Dr. W. Knowsley Sibley read a paper advocating the formation of a Central Hospital Board for London before the meeting of the Westminster Division of the Metropolitan Counties Branch of the British Medical Association, which was held on Wednesday, March 13, at St. James's Vestry Hall, Piccadilly. He commenced by taking a rapid glance at a few of the more prominent points in the present "uncontrolled" hospital system, including the question of competition between hospitals for funds, students, and patients; the crowding of hospitals into the wealthier districts where they were not wanted and their absence from the poorer parts where they were sadly required, and the misuse of hospitals. Neither the interests of the public nor those of the medical profession were safeguarded under the present system. Many of the subscriptions received by hospitals were paid by business houses as a form of insurance, in order to provide for the unlimited free treatment of their employees in case of accident or illness. In like manner some private individuals gave their few guineas a year to the nearest hospital. He advocated the amalgamation of King Edward's Hospital Fund and the Hospital Sunday and Saturday Funds into one central body, holding that if these bodies were to amalgamate there would be a large saving in expense and a much greater power to control and supervise hospital expenditure. A strong central body could at once stop the abuse and misuse into which the hospitals had allowed themselves to drift, restore them to the place they should hold in the social organisation, and rescue them from their lamentable present position of pauperising agents. The public and the profession were tired of the everlasting special appeals for funds, and the hospitals must now be rescued from the hands of competing groups of irresponsible individuals, and from the narrow cliques of many of the medical lay committees, and placed under the proper control of a central and responsible authority. The first business of a Central Metropolitan Board should be to draw up a Hospitals and Charities Acts on somewhat parallel lines to the Public Companies Acts, which should define the legal position of members of charity committees and the responsibilities of all those holding fiduciary positions. The subscribing public had a right to know that the money given by it was not, through negligence and stupidity, wantonly squandered in a way that the law would not permit to be followed by any but a charitable institution. (Applause.) The following motion was moved and carried unanimously: "That in view of the mismanagement and abuse of hospitals, the Westminster Division considers the welfare of the public and the interests of the medical profession would be best obtained by the establishment of a Central Hospital Board for London." It was decided to call a special meeting at an early date to consider the resolution.

